# Trial‐History Effects During WCST in Treatment‐Resistant Schizophrenia

**DOI:** 10.1002/npr2.70151

**Published:** 2026-08-03

**Authors:** Ryo Sawagashira, Atsuhito Toyomaki, Hayato Watanabe, Naoki Hashimoto, Takahiro A. Kato

**Affiliations:** ^1^ Department of Psychiatry, Graduate School of Medicine Hokkaido University Sapporo Japan; ^2^ Health Care Center Hokkaido University Sapporo Japan; ^3^ Center for Information and Neural Networks, Advanced ICT Research Institute National Institute of Information and Communications Technology Suita Japan

**Keywords:** dynamic behavioral phenotype, executive function, treatment‐resistant schizophrenia, trial‐history effect, Wisconsin card sorting test

## Abstract

**Aim:**

Treatment‐resistant schizophrenia (TRS) is a clinically important subtype of schizophrenia, but its cognitive characteristics remain incompletely understood. The Wisconsin Card Sorting Test (WCST) is widely used to assess executive dysfunction in schizophrenia, although conventional summary analyses may overlook trial‐by‐trial behavioral adaptation. This study aimed to examine whether recent trial outcome modulates subsequent responding differently between TRS and non‐TRS patients during the WCST.

**Methods:**

We analyzed WCST data from 41 outpatients with schizophrenia, including 13 patients with TRS and 28 with non‐TRS. Conventional subject‐level indices, including perseveration rate and outcome‐dependent reaction time (RT) adjustment, were first compared between groups. We then performed trial‐level linear mixed‐effects analyses using log‐transformed RT as the dependent variable, with previous trial correctness, treatment resistance, their interaction, age, and sex as fixed effects. Additional sensitivity analyses included models with by‐subject random slopes for previous trial correctness and analysis in an age‐matched sample.

**Results:**

Conventional summary analyses showed no significant group differences in perseveration rate or RT measures. In the primary model, previous trial correctness significantly modulated subsequent RT, and the interaction between previous trial correctness and TRS suggested a group difference in outcome‐dependent RT modulation. Specifically, the RT difference between trials following error and correct responses appeared smaller in TRS patients than in non‐TRS patients. However, sensitivity analyses using by‐subject random slopes and an age‐matched sample showed similar directions and magnitudes of the interaction but did not reach statistical significance.

**Conclusion:**

Conventional WCST summary analyses did not distinguish TRS from non‐TRS patients, whereas trial‐level modeling provided preliminary evidence for possible alteration of outcome‐dependent response adjustment in TRS. These findings suggest that dynamic behavioral phenotypes may provide a useful complement to standard neuropsychological indices in characterizing cognitive heterogeneity in schizophrenia, but replication in larger samples is needed.

## Abstract 2

In the WCST, participants must use feedback from each trial to update their subsequent choices. Trial‐level analysis revealed that previous‐trial correctness modulated reaction time, with a weaker post‐error slowing‐like effect in TRS compared with non‐TRS participants. These findings suggest that trial‐history‐dependent response adjustment may represent a dynamic behavioral phenotype of TRS.

## Graphical abstract

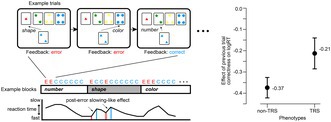

## 1 Introduction

Patients with schizophrenia commonly exhibit impairments in executive function, which contribute substantially to poor functional outcome [1]. The Wisconsin Card Sorting Test (WCST) has been widely used to assess executive dysfunction in schizophrenia, particularly cognitive inflexibility, impaired rule maintenance, and perseverative responding [2, 3]. Importantly, successful WCST performance requires not only maintaining an abstract sorting rule but also continuously using feedback from recent trials to guide subsequent choices [4]. Thus, trial‐by‐trial adaptation to positive and negative feedback is a central cognitive process underlying WCST performance.

Cognitive inflexibility is one of the most robust neuropsychological findings in schizophrenia, but schizophrenia is highly heterogeneous, and it remains unclear whether distinct cognitive phenotypes can be identified within this diagnostic category [5]. This question may be especially relevant in treatment‐resistant schizophrenia (TRS), a clinically important subtype associated with poorer clinical and functional outcomes as well as marked heterogeneity [6, 7]. Previous studies have suggested that patients with TRS may show greater neurocognitive impairment than those with non‐TRS [8]. However, most prior studies have evaluated WCST performance using conventional subject‐level summary indices, such as total errors, perseverative errors, or number of categories achieved [9, 10]. Although these aggregate measures capture overall task performance, they provide limited information about how patients dynamically adjust their behavior after recent success or failure.

Therefore, a trial‐level analysis of WCST performance may capture cognitive alterations that are obscured by conventional summary measures. In particular, examining how the outcome of the immediately preceding trial influences the next response allows us to assess feedback‐guided behavioral adjustment during the WCST. In the present study, we examined whether recent trial outcome modulates subsequent responding differently between patients with TRS and non‐TRS.

## 2 Methods

### 2.1 Participants and Data Collection

This study was approved by the Local Ethics Committee of Hokkaido University (approval number: 024‐0326). We analyzed WCST data from 41 outpatients with schizophrenia assessed at Hokkaido University Hospital between April 1, 2019, and October 31, 2025, including 13 patients with TRS and 28 with non‐TRS. TRS was operationally defined as a clinical diagnosis of treatment resistance made by board‐certified psychiatrists in patients receiving clozapine because of inadequate response or poor tolerability to prior antipsychotic treatment. To characterize the TRS definition, we reviewed the available medical records regarding clozapine initiation and prior antipsychotic exposure. Because this was a retrospective study, detailed information on the duration of each individual prior antipsychotic trial was not systematically available. The present study was derived from a broader neurocognitive assessment dataset. A previous study using the same broader dataset analyzed patients with bipolar disorder and major depressive disorder [10], whereas the present study focused on patients with schizophrenia and trial‐level WCST performance.

### 2.2 Summary‐Level Analysis

A computerized Japanese Keio University version of the WCST was used in this study [11]. This task assesses cognitive flexibility and set‐shifting. In the present study, perseveration rate was calculated using the Milner‐type definition, which indicates persistent responding according to a previously correct sorting principle. To quantify post‐error changes in reaction time (RT) at the subject level, RTs were first z‐scored within each participant to reduce the influence of individual differences in overall response speed. For each participant, we then calculated the mean z‐scored RT separately for trials following error or correct responses. The participant‐level index of post‐error RT adjustment was defined as the difference between these means, calculated as post‐error RT minus post‐correct RT. Group differences in this index were subsequently examined.

### 2.3 Trial‐Level Analysis

To retain trial‐history information, we then fitted a trial‐level linear mixed‐effects model with trial‐by‐trial reaction times as the dependent variable. Mixed‐effects modeling is well suited to RT data because it preserves trial‐level variation that may be obscured by subject‐level aggregation [12]. Log transformation was used because trial‐level RT distributions are typically right‐skewed, and this transformation made the outcome more appropriate for linear mixed‐effects modeling. Individual differences in overall response speed were modeled by the subject‐specific random intercept. The primary model was specified as follows:

$$\text{logRT}\sim \text{PrevCorrect}\times \mathrm{TRS}+\mathrm{Age}+\mathrm{Sex}+\left(1\;|\;\text{Subject}\right)$$

where PrevCorrect indicated whether the immediately preceding trial was correct or incorrect, TRS indicated treatment‐resistant status, and Subject was included as a random intercept. The key term of interest was the PrevCorrect × TRS interaction, which tested whether the effect of previous trial correctness on subsequent RT differed between TRS and non‐TRS patients.

As sensitivity analyses, we additionally fitted models allowing the effect of previous trial correctness to vary across subjects. Specifically, we fitted a random‐intercept/random‐slope model:

$$\text{logRT}\sim \text{PrevCorrect}\times \mathrm{TRS}+\mathrm{Age}+\mathrm{Sex}+\left(1+\text{PrevCorrect}\;|\;\text{Subject}\right)$$

Moreover, to examine the potential influence of age differences between groups, we repeated the primary trial‐level model in an age‐matched sample.

### 2.4 Statistical Analysis

Group comparisons were conducted using independent‐samples *t*‐tests and chi‐squared tests, with a significance threshold of *α* = 0.05. All statistical analyses were performed using MATLAB R2025.

## 3 Results

Demographics and WCST summary measures are summarized in Table 1. Compared with the non‐TRS group, the TRS group was younger and received higher chlorpromazine‐equivalent antipsychotic doses, whereas illness duration was longer in the non‐TRS group.

**TABLE 1:** Demographics and WCST summary measures in TRS and non‐TRS.

	Non‐TRS	TRS	*p*
Number	28	13	
Sex	Male 18/Female 10	Male 6/Female 7	0.32^a^
Age	39.93 ± 12.13	26.38 ± 5.14	**< 0.001** ^b^
Illness duration (days)	5058.39 ± 3652.90	1823.08 ± 1283.61	**< 0.01** ^b^
CP eq (mg/day)	517.89 ± 297.63	736.54 ± 350.98	**0.045** ^b^
DZP eq (mg/day)	11.08 ± 21.86	0.15 ± 0.22	0.08^b^
Clozapine (mg/day)	—	332.70 ± 118.80	—
Perseveration rate	0.06 ± 0.08	0.10 ± 0.13	0.25^b^
RT difference after correct vs. error trials (z‐scored)	0.80 ± 0.51	0.62 ± 0.55	0.30^b^

*Note:* CP eq doses included clozapine doses; RT difference was calculated as the mean reaction time after error trials minus the mean reaction time after correct trials. Z‐scored RT measures were computed within each participant. Bold values indicate *p* < 0.05.

Abbreviations: CP eq, chlorpromazine equivalent; DZP eq, diazepam equivalent; TRS, treatment‐resistant schizophrenia.

^a^ Chi square test.

^b^ Unpaired *t*‐test.

Among the 13 patients with TRS, clozapine was initiated because of inadequate response to prior antipsychotic treatment in 10 patients, poor tolerability in 2 patients, and both inadequate response and poor tolerability in 1 patient. The number of prior antipsychotics ranged from 2 to 8. For all patients whose clozapine initiation was related to inadequate response, at least two prior antipsychotic treatments met the dose criterion of chlorpromazine‐equivalent doses of ≥ 600 mg/day. The mean duration from illness onset to clozapine initiation was 978 days (SD = 856 days).

Perseveration rate did not significantly differ between groups, and neither RT following correct trials nor RT following error trials showed a significant between‐group difference. An additional age‐matched sensitivity analysis was conducted using 13 non‐TRS and 13 TRS patients to examine the potential influence of age differences between groups. Demographic and clinical characteristics of the matched sample are provided in Table S1. In this matched sample, the overall direction of group differences in conventional measures was preserved, although no significant between‐group differences were observed.

**FIGURE 1:**
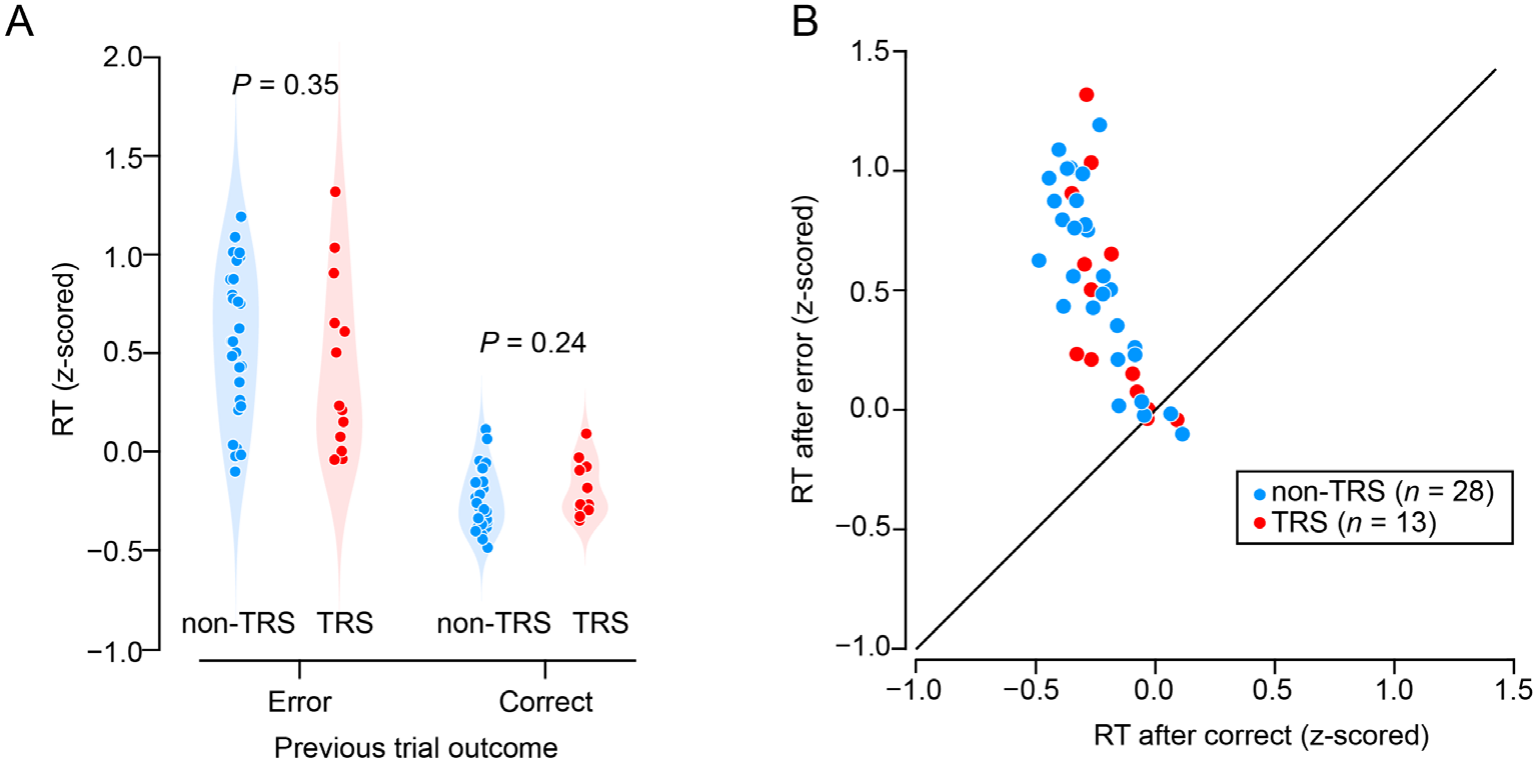
Reaction time distributions after correct and error trials in patients with schizophrenia. (A) Violin plots with individual data points showing z‐scored reaction times (RTs) in trials following error and correct responses in non‐treatment‐resistant schizophrenia (non‐TRS) and treatment‐resistant schizophrenia (TRS). No significant differences were observed in RT after error trials or RT after correct trials. (B) Scatter plot showing the relationship between RT after correct trials and RT after error trials across individuals. Each point represents one participant. Blue indicates non‐TRS patients and red indicates TRS patients.

As shown in Figure 1A, z‐scored RT distributions overlapped substantially between groups for both trial types. Figure 1B shows that most data points lay above the unity line, indicating that responses were generally slower after error trials than after correct trials across subjects. The distributions of TRS and non‐TRS patients appeared largely overlapping in this subject‐level plot.

**FIGURE 2:**
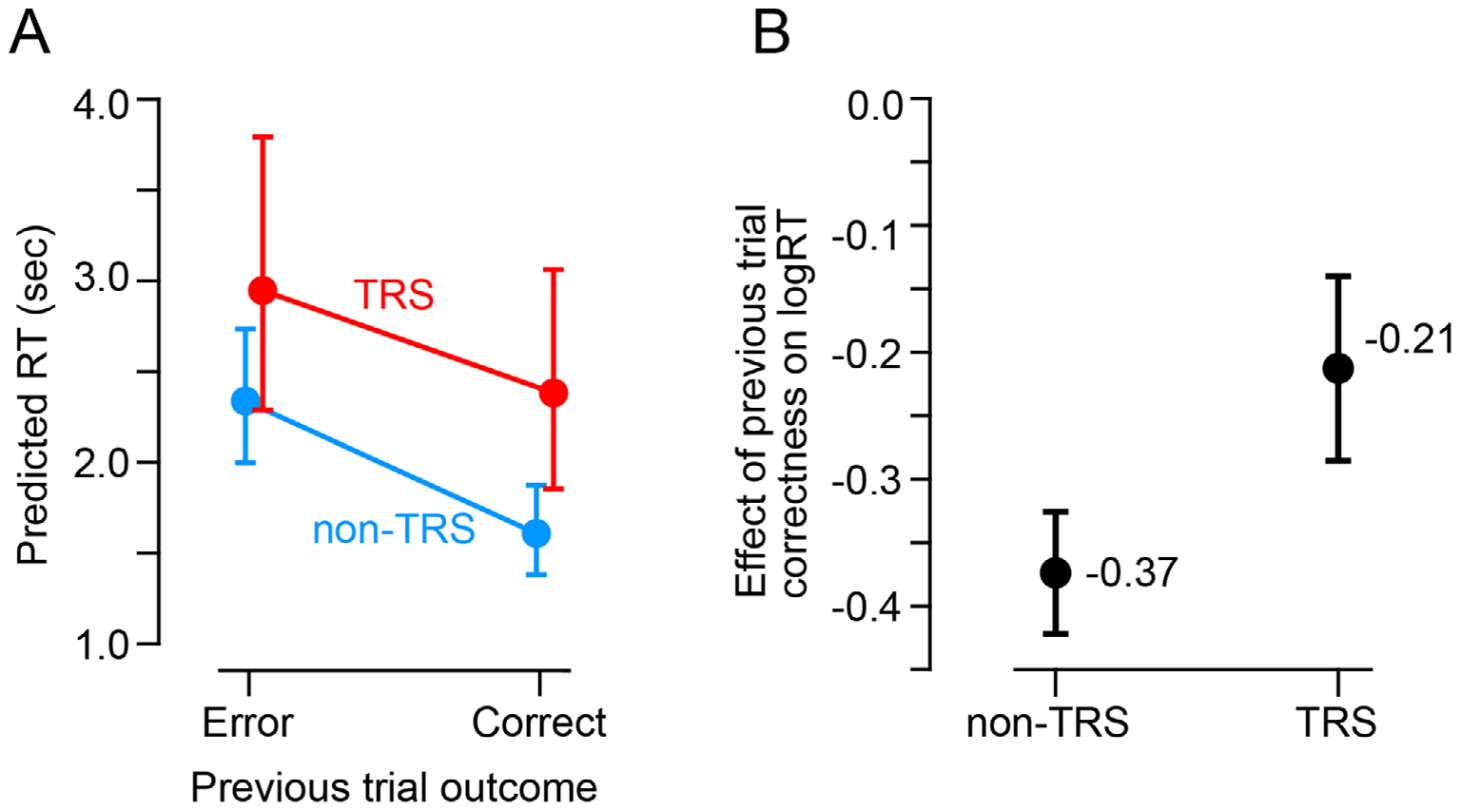
Trial‐level effects of previous trial outcome on reaction time in non‐TRS and TRS patients. (A) Predicted reaction times estimated from the linear mixed‐effects model according to previous trial outcome. RT shortening after correct trials was greater in non‐TRS patients and attenuated in TRS patients, consistent with a significant previous trial correctness × TRS interaction (*β* = 0.16, *p* = 2.97 × 10^−4^). (B) Group‐wise simple effects of previous trial correctness estimated from the same model. More negative values indicate greater RT shortening after correct than after error trials. Error bars represent 95% confidence intervals.

Taken together, conventional subject‐level analyses revealed no significant differences between TRS and non‐TRS patients, although responses tended to be slower after error trials than after correct trials across participants (post‐error slowing‐like effect). In contrast, in the primary trial‐level mixed‐effects model, previous trial correctness showed a significant main effect, indicating faster responses after correct trials than after error trials. Detailed coefficients of the primary linear mixed‐effects model are presented in Table 2. The PrevCorrect × TRS interaction was also significant in the primary model, whereas the main effect of TRS was not. As shown in Figure 2A, the RT difference between trials following error and correct responses was larger in non‐TRS patients and smaller in TRS patients. Group‐wise simple‐effects estimates from the same model also showed a larger effect of previous trial correctness in non‐TRS than in TRS patients (Figure 2B). These findings suggest that treatment resistance may be associated not with generalized slowing, but with altered outcome‐dependent RT modulation. We then examined the robustness of the PrevCorrect × TRS interaction using alternative model specifications. The PrevCorrect × TRS interaction showed a similar direction and magnitude in both the random‐intercept/random‐slope model (*β* = 0.16, SE = 0.08, *t* = 1.88, *p* = 0.06 in Table S2) and the age‐matched analysis (*β* = 0.10, SE = 0.05, *t* = 1.88, *p* = 0.06 in Table S3), although neither reached conventional statistical significance.

**TABLE 2:** Results of the linear mixed‐effects model for reaction time.

	Estimate	SE	*t*‐value	*p*
Error (prev. trial) (reference)	0.00	—	—	—
Correct (prev. trial)	−0.37	0.03	−15.27	**6.29 × 10** ^**−50**^
Non‐TRS (reference)	0.00	—	—	—
TRS	0.23	0.14	1.64	0.10
Correct (prev. trial) × TRS	0.16	0.04	3.62	**2.97 × 10** ^**−4**^
Age	0.02	5.29 × 10^−3^	3.61	**3.14 × 10** ^**−4**^
Male (reference)	0.00	—	—	—
Female	0.02	0.11	0.17	0.86

*Note:* The dependent variable was log‐transformed reaction time. Reaction times were log‐transformed before fitting the linear mixed‐effects model to reduce right‐skewness. Bold values indicate *p* < 0.05.

## 4 Discussion

The present study examined whether trial‐history effects during the WCST differed between TRS and non‐TRS patients. Conventional subject‐level WCST indices, including perseveration rate and mean RTs following correct or error trials, did not significantly differ between groups. In contrast, the primary trial‐level mixed‐effects model suggested a group difference in how previous trial outcome modulated subsequent RT. Specifically, the RT difference between trials following error and correct responses appeared smaller in TRS patients than in non‐TRS patients. However, this interaction did not reach conventional statistical significance in sensitivity analyses using by‐subject random slopes or in the age‐matched sample. Therefore, the present finding should be interpreted as preliminary evidence for possible alteration of outcome‐dependent RT modulation in TRS.

The main implication of this finding is that cognitive differences related to treatment resistance may be expressed in the temporal dynamics of behavior, even when conventional summary analyses do not show clear group differences. In the present sample, patients with TRS did not show generalized slowing in the primary model. Rather, the group difference appeared in the extent to which RT was modulated by the immediately preceding trial outcome. This pattern can be described as attenuated post‐error slowing, or equivalently as a smaller RT difference between post‐error and post‐correct trials, in the TRS group. In the context of the WCST, this pattern may reflect reduced stabilization of an effective response mode after correct trials, or a broader alteration in adaptive control processes that support efficient behavioral adjustment [4, 13]. This interpretation is consistent with prior work suggesting that WCST deficits in schizophrenia are related not only to overall executive impairment, but also to the online use of feedback and working‐memory‐related processes [14, 15]. Future studies should therefore examine whether neurophysiological abnormalities in TRS are more closely associated with dynamic behavioral phenotypes than with conventional summary scores.

The absence of significant group differences in conventional WCST indices should be interpreted cautiously. Summary measures such as perseveration rate and mean RT collapse across heterogeneous trial contexts and may therefore be relatively insensitive to subtle but systematic trial‐history effects. In addition, the present sample may not fully represent the broader TRS population because all participants were able to complete the WCST; patients with severe cognitive impairment, poor task engagement, or marked motivational difficulties may have been underrepresented. Moreover, because all patients in the TRS group were receiving clozapine, the observed pattern may reflect not only clinical characteristics of treatment resistance but also the effects of clozapine treatment. Clozapine may have improved psychotic symptoms, reduced behavioral disorganization, or increased task engagement, thereby attenuating group differences in conventional WCST performance. However, because this study was cross‐sectional and did not include WCST data before clozapine initiation, the effects of clozapine could not be separated from the characteristics of TRS itself.

## 5 Limitations

Several limitations should be acknowledged. First, the sample size was modest, particularly in the TRS group, and the findings should therefore be regarded as preliminary. Although the PrevCorrect × TRS interaction showed a consistent direction across sensitivity analyses, it did not reach conventional statistical significance in the random‐intercept/random‐slope model or in the age‐matched sample. A larger sample is needed to obtain more precise estimates and to determine whether the observed group difference in outcome‐dependent RT modulation reflects a reliable effect or sampling variability. Second, TRS was operationally defined in clozapine‐treated patients on the basis of clinical diagnosis and prior inadequate response or poor tolerability to antipsychotic treatment. Although we confirmed available information regarding clozapine initiation and prior antipsychotic exposure, detailed information on the duration of each individual prior antipsychotic trial was not systematically available. Therefore, we could not fully reapply a standardized research algorithm for TRS to each participant. In addition, medication‐related effects cannot be fully separated from treatment resistance itself. Third, the groups differed in age, illness duration, and antipsychotic dose, all of which may have influenced performance. Although age and sex were included in the model and an age‐matched sensitivity analysis was conducted, residual confounding remains possible. In particular, clozapine treatment, illness chronicity, medication dose, and other clinical background factors may have contributed to the observed behavioral patterns. Fourth, the present analysis focused on RT and did not examine other trial‐level behavioral outcomes, such as perseverative responses, switching behavior after negative feedback, or win‐stay/lose‐shift patterns. Such analyses could provide complementary information about feedback‐based behavioral updating beyond RT and should be examined in future studies.

## 6 Conclusion

In conclusion, conventional WCST summary analyses did not detect significant differences between TRS and non‐TRS patients, whereas trial‐level mixed‐effects modeling provided preliminary evidence for possible alteration of outcome‐dependent RT modulation in TRS. The direction and approximate magnitude of the key interaction were preserved across sensitivity analyses, but the statistical evidence was weakened after accounting for individual differences in within‐subject effects and age matching. These findings suggest that dynamic behavioral phenotypes may provide a useful complement to standard neuropsychological indices in characterizing cognitive heterogeneity in schizophrenia, but replication in larger samples is needed.

## Author Contributions

R.S. contributed to conceptualization, methodology, formal analysis, visualization, project administration, writing – original draft, and writing – review and editing. H.W. contributed to supervision and writing – review and editing. A.T. contributed to methodology, supervision, and writing – review and editing. N.H. contributed to supervision and writing – review and editing. T.A.K. contributed to supervision and writing – review and editing. All authors reviewed and approved the final manuscript.

## Funding

This work was supported by the Japan Agency for Medical Research and Development (AMED) (Grant Number: 26dk0307132s0203; Principal Investigator: Naoki Hashimoto). The funder had no role in the study design, data collection, analysis, or interpretation, manuscript preparation, or the decision to submit for publication.

## Ethics Statement

This study was approved by the Local Ethics Committee of Hokkaido University (approval number: 024‐0326) and was conducted in accordance with the Declaration of Helsinki and its later amendments.

## Consent

In accordance with institutional guidelines for retrospective studies, written informed consent was waived, and information about the study was publicly disclosed to allow patients the opportunity to opt out.

## Conflicts of Interest

The authors declare no conflicts of interest.

## Supporting information


**Table S1:** Demographics and WCST summary measures in age‐matched population.
**Table S2:** Results of the linear mixed‐effects model for reaction time with by‐subject random slopes.
**Table S3:** Results of the linear mixed‐effects model for reaction time in age‐matched population.

## Data Availability

The datasets generated and analyzed during the current study are not publicly available because they contain sensitive clinical data from human participants, including retrospective clinical assessment data, and public data sharing was not covered by the original consent procedures. Because some participants could not be re‐contacted for study‐specific consent, the study was conducted under an ethics‐committee‐approved opt‐out framework. De‐identified data may be available from the corresponding author on reasonable request, subject to approval by the relevant ethics committee.
