# Supplementary material for: Trial‐History Effects During WCST in Treatment‐Resistant Schizophrenia
Source: Neuropsychopharmacol Rep. 2026 Aug 3;46(3):e70151. doi: 10.1002/npr2.70151 (PMC13434216; doi:10.1002/npr2.70151)
Supplement: Supplementary file 1 [file NPR2-46-e70151-s001.docx]

**Supplementary Table 1. Demographics and WCST summary measures in age-matched population**

|  | non-TRS | TRS | *P*-value |
| --- | --- | --- | --- |
| Number | 13 | 13 |  |
| Sex | male 9 / female 4 | male 6 / female 7 | 0.43^†^ |
| Age | 28.69 ± 5.78 | 26.38 ± 5.14 | 0.29^§^ |
| Illness duration (days) | 2360.46 ± 1921.70 | 1823.08 ± 1283.61 | 0.41^§^ |
| CP eq (mg/day) | 504.87 ± 202.22 | 736.54 ± 350.98 | 0.05^§^ |
| DZP eq (mg/day) | 9.64 ± 19.21 | 0.15 ± 0.22 | 0.08^§^ |
| Clozapine (mg/day) | − | 332.70 ± 118.80 | − |
| Perseveration rate | 0.05 ± 0.10 | 0.10 ± 0.13 | 0.28^§^ |
| RT difference after correct vs error trials (z-scored) | 0.90 ± 0.16 | 0.79 ± 0.22 | 0.14^§^ |

†Chi square test; §unpaired t-test; TRS: treatment-resistant schizophrenia;

CP eq: chlorpromazine equivalent; DZP eq: diazepam equivalent;

CP eq doses included clozapine doses;

RT difference was calculated as the mean reaction time after error trials minus the mean reaction time after correct trials. Z-scored RT measures were computed within each participant.

**Supplementary Table 2. Results of the linear mixed-effects model for reaction time with by-subject random slopes**

|  | estimate | se | *t*-value | *P*-value |
| --- | --- | --- | --- | --- |
| Error (prev. trial) (reference) | 0.00 | − | − | − |
| Correct (prev. trial) | −0.39 | 0.05 | −8.11 | **8.91×10^−16^** |
| non-TRS (reference) | 0.00 | − | − | − |
| TRS | 0.24 | 0.14 | 1.72 | 0.09 |
| Correct (prev. trial) × TRS | 0.16 | 0.08 | 1.88 | 0.06 |
| age | 0.02 | 0.01 | 3.72 | **2.04×10^−4^** |
| male (reference) | 0.00 | − | − | − |
| female | −0.05 | 0.11 | −0.49 | 0.62 |

**Note.** The dependent variable was log-transformed reaction time. Reaction times were log-transformed before fitting the linear mixed-effects model to reduce right-skewness.

**Supplementary Table 3. Results of the linear mixed-effects model for reaction time in age-matched population**

|  | estimate | se | *t*-value | *P*-value |
| --- | --- | --- | --- | --- |
| Error (prev. trial) (reference) | 0.00 | − | − | − |
| Correct (prev. trial) | −0.31 | 0.04 | −8.02 | **2.57×10^−15^** |
| non-TRS (reference) | 0.00 | − | − | − |
| TRS | 0.25 | 0.16 | 1.56 | 0.12 |
| Correct (prev. trial) × TRS | 0.10 | 0.05 | 1.88 | 0.06 |
| age | 0.02 | 0.02 | 1.27 | 0.21 |
| male (reference) | 0.00 | − | − | − |
| female | −0.02 | 0.17 | −0.12 | 0.91 |

**Note.** The dependent variable was log-transformed reaction time. Reaction times were log-transformed before fitting the linear mixed-effects model to reduce right-skewness.
